# Hotspots in a cold land‐reported cases of rabies in wildlife and livestock in Mongolia from 2012–2018

**DOI:** 10.1111/zph.12954

**Published:** 2022-05-18

**Authors:** Graham A. Matulis, Doniddemberel Altantogtokh, Paul M. Lantos, Jordan H. Jones, Rachel N. Wofford, Mark Janko, Nyamdorj Tsogbadrakh, Tserendovdon Bayar, Sainkhuu Ganzorig, Bazartseren Boldbaatar, B. Katherine Poole‐Smith, Jeffrey Hertz, Jodi Fiorenzano, Michael E. von Fricken

**Affiliations:** ^1^ Department of Global and Community Health George Mason University Fairfax Virginia USA; ^2^ National Center for Zoonotic Diseases Ulaanbaatar Mongolia; ^3^ Duke University School of Medicine Durham North Carolina USA; ^4^ Duke Global Health Institute Durham North Carolina USA; ^5^ University of Washington Seattle Washington USA; ^6^ General authority for veterinary services Ulaanbaatar Mongolia; ^7^ School of Veterinary Medicine Mongolian University of Life Sciences Ulaanbaatar Mongolia; ^8^ Department of Entomology Armed Forces Institute of Medical Sciences (AFRIMS) Bangkok Thailand; ^9^ Naval Medical Research Unit TWO (NAMRU‐2) Sembawang Singapore

**Keywords:** Central Asia, Mongolia, public health, rabies, rabies virus, viral zoonoses

## Abstract

The epidemiological profile of rabies virus within Mongolia remains poorly characterized despite 21,302 domestic animal cases being reported between 1970–2005. This lack of knowledge is particularly concerning given that roughly 26% of the population lives a pastoral herding lifestyle and livestock production contributes up to 18% of Mongolia's total gross domestic product (GDP). The gaps in knowledge of the rabies disease ecology within Mongolia combined with the lack of routine vaccination of domestic animals and wildlife poses a significant threat to the more than 60 million heads of livestock within Mongolia. Animal rabies case data from the General Authority for Veterinary Services and National Center for Zoonotic Diseases were used in this study. Each data point included year of report, an animal descriptor, geographic coordinates and the aimag (province) of origin. A total of 2,359 animal rabies cases were reported between 2012–2018. Cattle were the most commonly reported animal overall (861 cases), followed by goats (268), sheep (251) and dogs (221) within the domestic animal category. Red foxes were responsible for most reported wildlife cases (317) followed by wolves (151). Most rabid animals were reported in the Khuvsgul, Uvurkhangai and Govi‐Altai aimags, and a positive correlation was found between livestock numbers per soum and the number of rabies cases reported. Rabies poses a significant threat to the Mongolian economy and the health of human and animal populations within Mongolia. The close association of the nomadic pastoralists with both domestic animals and wildlife represents a significant threat for disease emergence and necessitates studies that describe the ecology of rabies, which may threaten these populations.

**Impacts**

- Two thousand three hundred fifty nine rabies cases were reported in domestic animals (1,872 cases) and wildlife (487 cases) within Mongolia between 2012–2018.
- Rabies virus was most commonly reported in cattle (861 cases), followed by red foxes (317 cases), goats (268 cases) and sheep (251 cases). The aimag with the most rabies case reports was Khuvsgul (328 cases), followed by Uvurkhangai (272 cases) and Govi‐Altai (266 cases).
- There was a positive correlation between the number of livestock per soum and the number of reported rabies cases. No association was seen between human population density and the number of reported rabies cases in livestock or wildlife.

## 1 INTRODUCTION

Rabies is a zoonotic disease, which is almost always fatal among individuals who develop symptoms (Hutter et al., 2018). The disease is caused by the rabies virus, which is typically transmitted through saliva, often during an animal bite (Hutter et al., 2018). Globally, 50,000–60,000 people die each year from rabies, with deaths attributed to ineffective reservoir animal control and inadequate access to pre‐ and post‐exposure prophylaxis (PEP) (Boldbaatar et al., 2010; Deviatkin et al., 2017; Hutter et al., 2018; Jemberu et al., 2013; Jibat et al., 2016; Zhu et al., 2015). While rabies reservoir species differ geographically, 90% of human cases worldwide can be attributed to domestic or stray dogs (Feng et al., 2015; Jemberu et al., 2013; Shao et al., 2011; Tao et al., 2019). The global economic impact of rabies is estimated at $8.6 billion annually, with 6% resulting from livestock losses (Food and Agriculture Organization, 2017). The economic impact as it relates to human rabies cases is equivalent to 2 million disability‐adjusted life years (DALYs) or $4 billion (Deviatkin et al., 2017; Jemberu et al., 2013). Prevention and control strategies within the African and Asian regions alone costs $500–583 million annually (Jemberu et al., 2013; Sambo et al., 2013).

Mongolia is particularly vulnerable to damage from uncontrolled zoonotic disease transmission. An estimated 26% of the population lives a nomadic pastoralist lifestyle, and 37% of households own livestock (Barnes et al., 2020; Odontsetseg et al., 2009). The country's agriculture sector accounts for 21.7% of its GDP, 84.7% of which comes from livestock (Odontsetseg et al., 2009). According to the Mongolian Statistical Information Service, 67 million heads of livestock were present within Mongolia in 2020 (www.1212.mn).

Like many zoonotic diseases, the epidemiological profile of rabies within Mongolia is not well characterized, even though cases have been recorded since 1950 (Tuvshintulga et al., 2015). Only 34 human cases have been reported in the last 30 years, although roughly 2000 people receive PEP for rabies each year (Boldbaatar et al., 2010; Odontsetseg et al., 2009). The epidemiology of rabies within Mongolia is complicated by the low rates of immunization of both domestic animals and wildlife in Mongolia (Odontsetseg et al., 2009). Historically, red foxes (*Vulpes vulpes*) have been the most commonly reported rabid animal, followed by corsac foxes (*Vulpes corsac*), manul (*Otocolobus* spp.), dogs (*Canis lupus familiaris*) and wolves (*Canis lupus*) (Odontsetseg et al., 2009). From 1970–2005, 21,302 cases were reported in domestic animals, with cattle (*Bos* spp.), camels (*Camelus* spp.) and sheep (*Ovis* spp.) being the most commonly affected animals (Odontsetseg et al., 2009). Although wolves have been less commonly reported as having rabies within Mongolia, they are estimated to be the source of rabies infection in 73.1% of small ruminant cases, 12.6% of equine cases and 32.4% domestic canine cases (Odontsetseg et al., 2009). The enhanced contribution of rabies cases despite lower amounts of rabies reports may reflect both the reported higher frequency with which wolves prey on domestic animals and their widespread distribution across the country (Augugliaro et al., 2020; Lieb et al., 2021; Salvatori et al., 2021).

Improvements in rabies surveillance efforts that incorporate both wildlife and domestic animals would allow for more targeted and effective means of lowering rabies transmission (Ahmad et al., 2017; Brookes et al., 2019; Liu et al., 2016). This study sought to contribute to what is known about the disease ecology and geographic distribution of rabies cases reported within domestic and wild mammals of Mongolia through the use of positive‐only passive surveillance. This data, in the context of previous reports of rabies within Mongolia, can be used to generate hypotheses to predict the future impacts of this disease while also informing the development of new prevention and control strategies for rabies.

## 2 MATERIALS AND METHODS

### 2.1 Data sources

**FIGURE 1:**
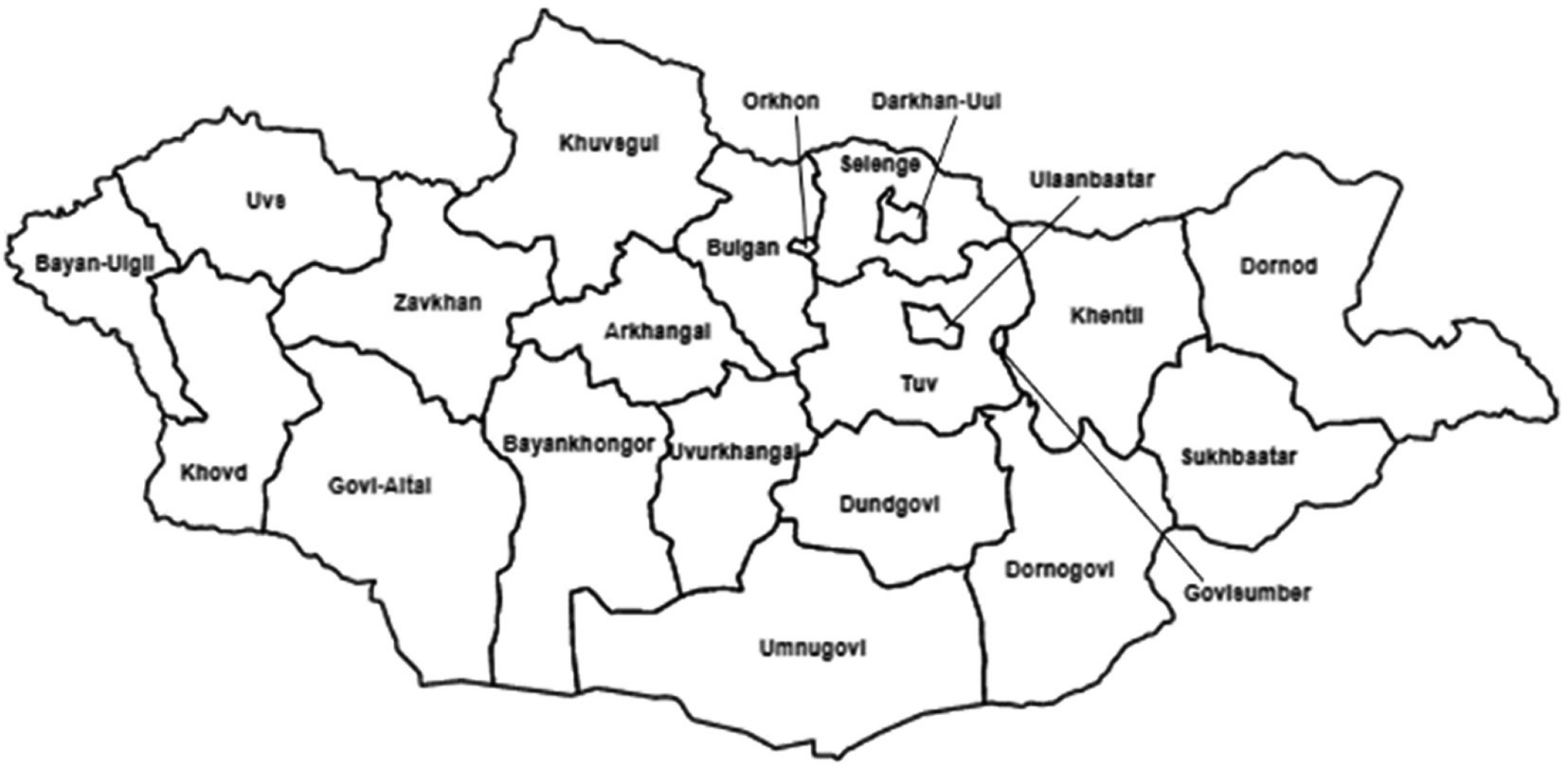
Administrative map of Mongolian Aimags

Data was acquired from the General Authority for Veterinary Services and the National Center for Zoonotic Diseases (NCZD). The NCZD actively investigates and confirms reports of rabies across Mongolia. The General Authority for Veterinary Services receives data from both the NCZD and veterinarians that report positive rabies cases. All rabies testing in Mongolia relies upon direct fluorescent antibody assays, which is the standard for routine rabies determination. Testing would occur when rabies was suspected due to clinical signs or suspected exposure. Each report included the animal's geographic coordinates, aimag (province) of origin, year of report and type of animal. Cases reported between 2012–2018 from all 21 aimags (Figure 1) and the independent provincial municipality Ulaanbaatar were included. Animal groups represented within the study included badgers (*Meles* spp.), camels, cats (*Felis catus*), cattle, corsac foxes, deer (Family: Cervidae), dogs, red foxes, goats (*Capra* spp.), horses (*Equus* spp.), lynx (*Lynx* spp.), manuls, sheep and wolves. Population and livestock density data were obtained from the Mongolian National Statistics Office (https://opendata.1212.mn/en/doc). The use of positive‐only passive surveillance can allow for an assessment of the locations from which most reports of rabies are coming from as well as the relative contribution from each animal group. These results may allow for a characterization of livestock animals that are most frequently infected by rabies and their location, while also allowing for an assessment of the rabid wildlife animals that are most frequently detected by humans. However, caution must be taken when trying to discern between increases in transmission intensity and increases in surveillance or case detection. It is important to note that the quality of positive‐only data can be influenced by various factors, such as whether clinical signs are observable in the affected animal, awareness of rabies by the persons reporting the disease and diagnostic test sensitivities (Hadorn et al., 2008).

Spatial analyses were conducted using ArcGIS (ESRI) and R 3.6.1 (www.r‐project.org). The coordinate location of each animal was projected in GIS. We conducted two spatial analyses: kernel density and linear regression. Kernel density uses a 2‐dimensional scanning radius to compute the density of rabies case distribution. This analysis was performed using the Kernel Density function in ArcGIS. Parameters for this tool included a 2,500 m^2^ output cell size and the default bandwidth.

The goal of our regression analysis was to evaluate how human population and livestock abundance contributed to the number of rabies cases reported in animals. Our regression analysis was a Bayesian model using the brms package in R and the Bayesian sampling program Stan (Bürkner, 2017; Carpenter et al., 2017). The units of analysis were the 339 soums (counties) in Mongolia. The outcome variable was the total count of rabies cases per soum (including both wild and domestic animals), linear predictor variables were number of humans and number of livestock per soum, and we used a Poisson likelihood function. To account for spatially correlated error, we constructed a neighbour matrix of the 339 soums in Mongolia using the spdep R package and used a Besag‐York‐Mollie 2 autocorrelation structure in our model specification (Riebler et al., 2016). Model comparison using the Watanabe‐Aikake information criterion (WAIC) strongly favoured this correlation structure.

## 3 RESULTS

In total, 1,667 reports equating to 2,359 animal rabies cases were included in the study. The average number of cases per report was 1.4 cases, with a range of 1–52 cases per report and only 35 reports having more than 5 cases. Apart from Darkhan, Govisumber, Orkhon and Selenge, all aimags reported cases in both domestic and wild mammals (Table 1). The highest case counts came from the Khuvsgul aimag (328, 13.9% of total animal cases), followed by Uvurkhangai (272, 11.5%) and Govi‐Altai (266, 11.3%). Cattle accounted for the most cases (861 cases, 36.5% of total animal cases) reported within the study timeframe, followed by red fox (317 cases, 13.4%), goat (268 cases, 11.4%) and sheep (251 cases, 10.6%). Red fox cases accounted for 65.1% of all rabies cases recorded in wild mammals. The four aimags reporting the most rabies cases all reported more than 30 rabies cases in red foxes. Wolves accounted for only 6.4% of all cases but represented 31% of wild mammal cases. The aimags reporting the most rabies cases overall were also the aimags reporting the highest amounts of cases in wolves. The highest proportion of rabid wolves (37.1%) was reported in Khuvsgul, which also reported the highest proportion of sheep (33.1%) and goat (23.1%) cases. Domestic dogs, which are most often associated with human rabies cases globally, only accounted for 9.4% of total animal cases (221 cases total), with 55.6% of reports coming from Govi‐Altai, Uvurkhangai, Bayankhongor and Khuvsgul. Of note, certain aimags such as Bayan‐Ulgii, Bulgan and Sukhbaatar reported high numbers of domestic animal cases, while also reporting low levels (≤5 cases) in foxes and wolves.

**TABLE 1:** Number of rabies cases by animal group and aimag, with a heat map colour

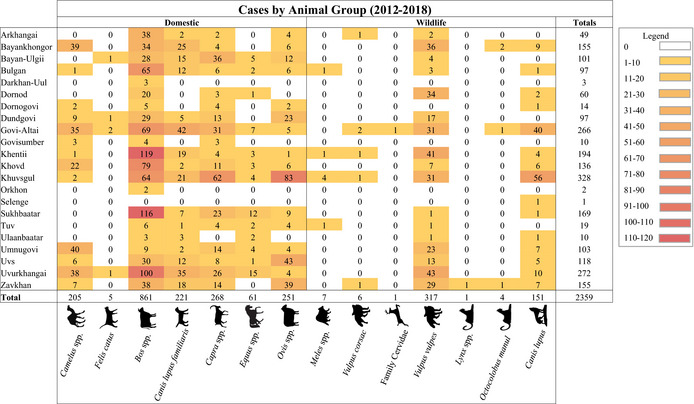

**FIGURE 2:**
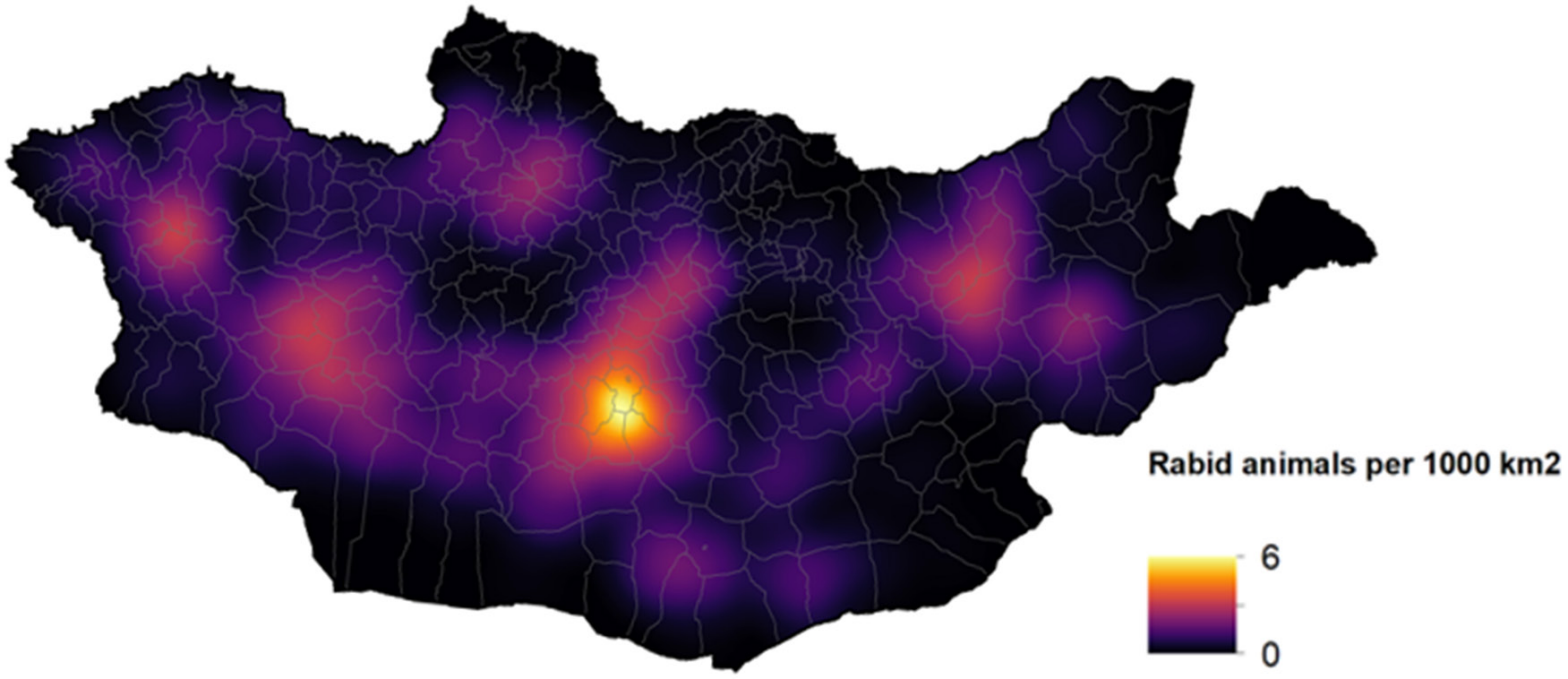
Kernel density model of rabies. Kernel model showing the density of rabies cases in domestic animals and wildlife per unit area within Mongolia

**FIGURE 3:**
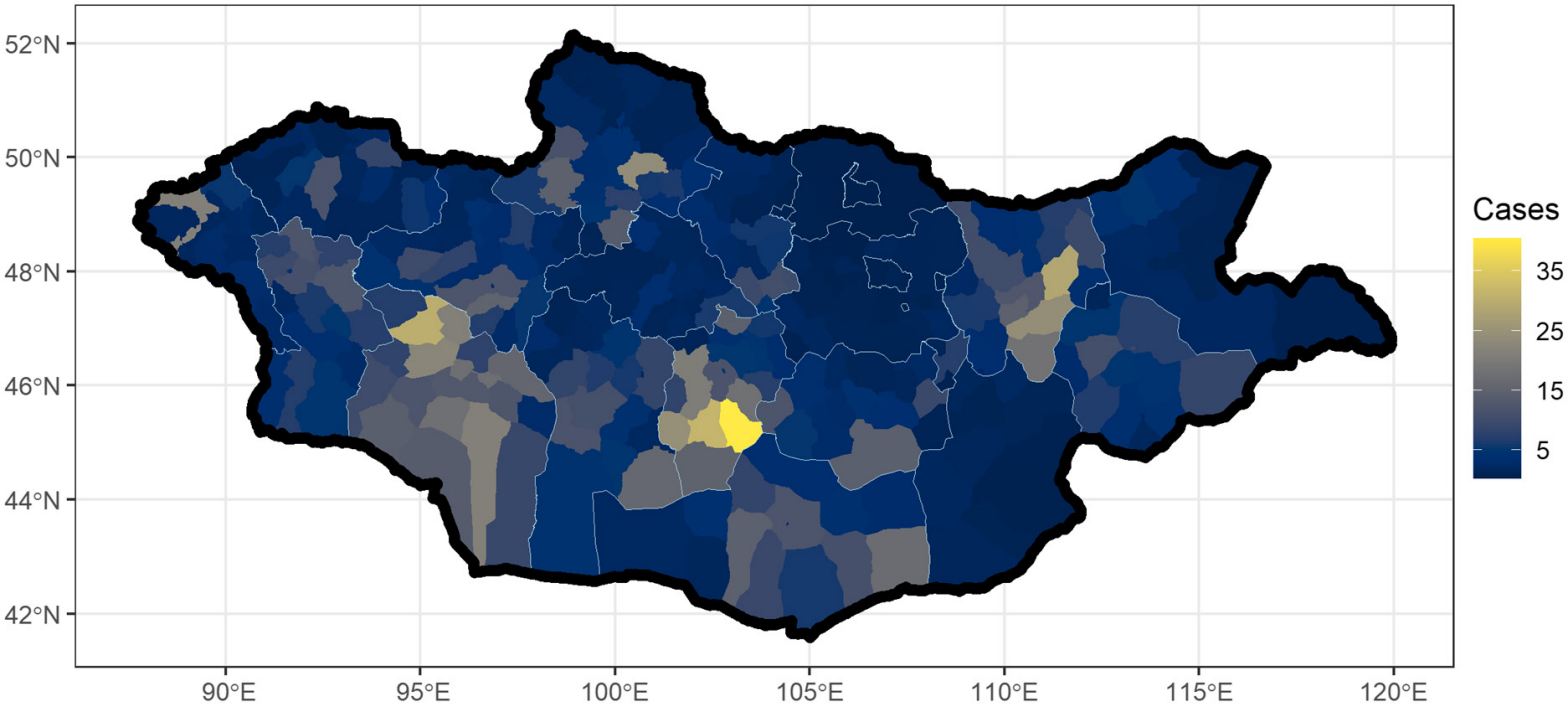
Regression plot adjusting density of rabies cases after adjusting for livestock spatial Poisson regression model, with the number of animal rabies cases predicted for the soums (counties) within Mongolia after adjusting for human and livestock populations

The density of rabies cases was distributed heterogeneously in Mongolia, with pockets of higher density seen in central and western Mongolia as well as isolated points in other areas of the country (Figure 2). It is important to note, that we are examining positive‐only data in this study, and that darker spots on this map are areas where we have no data to inform density, rather than an absence of rabies. The number of livestock per soum was directly correlated with the count of rabies cases. Every 1 standard deviation increase in livestock, corresponding to 90,341 animals, was associated with a 1.5‐fold increase in rabies cases (95% CI: 1.4–1.8, probability of positive association ~100%). The human population was not associated with the number of rabies cases (1.0, 95% CI: 0.8–1.2, probability of positive association 0.5). Although livestock populations were associated with rabies risk, the predicted geographic distribution of rabies cases was similar to the density map even after adjusting for livestock and human populations (Figure 3).

## 4 DISCUSSION

### 4.1 Mapping hotspots of rabies

We have found that animal rabies cases are heterogeneously distributed in Mongolia. The highest number of domestic animal cases were reported in Khuvsgul, Uvurkhangai and Govi‐Altai. In comparison, Odontsetseg et al. 2009 reported the highest livestock cases from 1996–2005 in Khuvsgul, Govi‐Altai, Khovd and Uvs (Odontsetseg et al., 2009). Mongolia has previously experienced patterns of rabies emergence in the east, followed by western movement of these rabies transmission foci throughout the country (Odontsetseg et al., 2009). Since all the western aimags reported over 100 cases during this timeframe, these results may demonstrate a continuation of this emergence pattern that has been seen in the past. However, because rabies cases were spatially associated with the distribution of livestock, the change in rabies distribution may correspond to unmeasured shifts in herding over this time.

Khuvsgul reported the most animal cases in this study (328), with the highest individual aimag case counts for sheep (83 cases), goats (62 cases) and wolves (56 cases). This finding is in agreement with Odontsetseg et al. 2009, who found that Khuvsgul had high case counts between 1996–2005, with a re‐emergence event occurring in 2000 (Odontsetseg et al., 2009). This indicates that Khuvsgul has continued to be an aimag with high levels of rabies activity, warranting future monitoring. Uvurkhangai reported the second most cases in this study, including 100 cattle cases and the highest amount of red fox and horse cases. While Uvurkhangai has reported a decreasing rabies burden since the 1970s, it reported the highest number of cases in wild carnivores between 1996–2005, possibly demonstrating that uncontrolled rabies transmission within wildlife has allowed for a persistently high case burden within the aimag (Odontsetseg et al., 2009). Of note, Govi‐Altai reported some of the highest case numbers in both this study and between 1996–2005 (Odontsetseg et al., 2009). The persistence of this region as a hotspot for reported rabies cases, despite the aimag having comparatively less intense livestock practices and a large amount of desert and mountain land cover, warrants surveillance and future studies of the transmission cycles occurring within this aimag (Erdenesan, 2016; Food and Agriculture Organization, 2017; Odontsetseg et al., 2009; Sheehy & Damiran, 2012). Relatively low case counts were reported from the Tuv, Darkhan, Dornogovi, Govisumber, Orkhon, Selenge and Ulaanbaatar aimags. While this might reflect a low incidence of rabies within these aimags, it could also indicate low levels of reporting or limited surveillance. It is important to note that Mongolia contains various ecosystems, including arid and semi‐arid Gobi regions in the south, vast steppe regions in the central region, Siberian forests to the north and natural barriers resulting from the Altai mountains in the west (Dugarsuren et al., 2011). Such ecological variations may have influences on the reporting and distribution of animal cases.

### 4.2 Potential reservoirs

Within Mongolia, the red fox, corsac fox, wolf and manul cat are all considered reservoir hosts of rabies (Odontsetseg et al., 2009). Red foxes, dogs and wolves were the most frequently reported carnivores in this study, with red foxes representing one of the top three most reported rabid animals in 10 aimags. In comparison, the dog and wolf represented 9.4% and 6.4% of total reported animal cases, respectively. This aligns with previous work, which concluded that red foxes are the most frequently detected rabid carnivore within Mongolia, and that dogs are more often reported to be rabid than wolves (Odontsetseg et al., 2009). However, frequency of rabies reports from an animal group does not necessarily reflect importance within disease transmission networks, as wolves have been reported as a significant source for infections in domestic animals throughout Mongolia, including 32.4% of dog rabies cases, despite low levels of rabies cases being reported for wolves (Odontsetseg et al., 2009). Of note, Sukhbaatar reported the second most cattle cases (116) in this study, although relatively few cases of rabies in dogs, red foxes and wolves were reported in this aimag. Such findings demonstrate a need for an active surveillance system that can both effectively detect rabies within more common hosts and identify reservoir hosts that are unique to certain aimags such as Sukhbaatar. Consistent surveillance in this eastern part of Mongolia is especially important, given previous reports of rabies circulation between Mongolia, Russia and China that has been linked to both natural animal movement and animal transportation as part of the fur trade (Liu et al., 2020; Liu et al., 2016; Tao et al., 2019; Yakovchits et al., 2021).

### 4.3 Risk to nomadic herders and the Mongolian population

Pastoral life in Mongolia includes extensive sharing of resources (i.e. water sources, manure used to fuel fires, shared areas for food preparation) with livestock animals, while also having regular contact with other domestic animals and wildlife (Barnes et al., 2017, 2020). These interactions increase the risk of the disease transmission among wildlife, domestic animals and nomadic pastoralists (Bawa et al., 2018). Barnes et al. 2020 found that while over 70% of Mongolian herders knew that diseases can be transmitted from animals to humans, only 36% knew of the disease risk from animal bites and scratches, demonstrating a major knowledge gap for this vulnerable population that is relevant to rabies (Barnes et al., 2020). The vulnerability of this population to diseases like rabies is further complicated by barriers to exposure response healthcare, such as distance to healthcare facilities and cost of post‐exposure prophylaxis (Sambo et al., 2013; Sreenivasan et al., 2019; Wild et al., 2019). Of note, the Uvurkhangai aimag, which had the second most animal cases in this study, reported 14.7% of the human rabies cases that occurred between 1970–2005 (Odontsetseg et al., 2009). In contrast, the Khuvsgul aimag, which reported the most cases in the current study, did not report any cases in the 1970–2005 timeframe (Odontsetseg et al., 2009). While the timeframes of these data differ, these findings highlight a need to improve the surveillance efforts for human rabies, to determine if there is any relationship between hotspots found for both animal and human rabies. In addition to the direct health impacts rabies can have on Mongolian populations such as nomadic pastoralists, losses in livestock can also economically impact populations and can be a threat to food security (Jemberu et al., 2013; Jibat et al., 2016). It has been observed that herders do not always report occurrences of low‐level morbidity or mortality among their animals in a timely manner, which may allow for the dissemination of various diseases throughout herds (Barnes et al., 2020). Additionally, while our study did not differentiate domestic animal cases by ownership or raising method, studies in other countries have shown higher rabies burdens in pastoral system herds (Ahmad et al., 2017; Brookes et al., 2019; Jibat et al., 2016; Zhu et al., 2011). These various factors illustrate how the health and wellbeing of the Mongolian population can be detrimentally impacted by uncontrolled transmission of zoonotic diseases, thus necessitating the establishment of an active surveillance system that works to detect diseases such as rabies.

## 5 LIMITATIONS

The analyses in this paper are based on passive surveillance data reported to the NCZD. Passive surveillance methods may overestimate cases of rabid livestock, with which humans have more contact, and underestimate cases in wildlife, or result in overestimations of rabies in wildlife carcasses that were tested under the fur industry. This limitation is supported by our finding that rabies cases were associated with the absolute number of livestock animals. Given the lack of active surveillance systems in place for rabies, differences observed between aimags may reflect variations in local capacity to send samples to centres capable of diagnosing rabies. Passive surveillance suffers from multiple drawbacks as it only captures cases with clear clinical presentations of rabies, may disproportionately result in testing and reporting of key wildlife species and major livestock groups, and relies heavily on local community awareness of rabies (Hadorn et al., 2008). Additionally, the use of positive‐only data cannot differentiate whether higher case counts are a result of increased transmission or indicative of regional differences in surveillance. Finally, we are unable to adjust findings based on wildlife distributions. Instead, this study lays a foundation for future active surveillance investigations of rabies in Mongolia.

An updated genetic characterization of rabies viruses detected within livestock and wildlife would hold value and potentially capture movement of rabies both within and outside of Mongolia. Surveillance of both domestic animals and wildlife should take into consideration any local vaccination practices in place, to determine the efficacy of such preventative measures in Mongolia. Future studies should characterize the context in which human rabies exposures occurred to identify both risk factors and better characterize the more relevant rabies reservoir host for human cases. Rabies continues to pose a significant threat to the economy and the health of human and animal populations within Mongolia. Nomadic pastoralists who share natural space with both domestic animals and wildlife are at an elevated risk of disease transmission and warrant further investigation.

## CONFLICT OF INTEREST

The authors have no conflicts of interest to declare.

## ETHICAL APPROVAL

This work is considered exempt as a secondary data analysis of confirmed rabies cases in domestic and wild animals, provided by the General Authority for Veterinary Services and the National Center for Zoonotic Diseases, Mongolia. No identifying information or PHI related to herders were included in the dataset that would require full ethical review. This data is publicly available upon request.

## Data Availability

The data that support the findings of this study are available from the corresponding author upon reasonable request.
